# Investigating the Predation Risk of Coastal Dolphins via the Presence of Shark Bite Scars Across Southeast Queensland, Australia

**DOI:** 10.1002/ece3.73691

**Published:** 2026-05-27

**Authors:** Georgina V. Hume, Alexis L. Levengood, Gemma L. Webster, Kathy A. Townsend, Victor M. Peddemors, Bonnie J. Holmes

**Affiliations:** ^1^ School of Science, Technology & Engineering University of the Sunshine Coast Sippy Downs Queensland Australia; ^2^ Marine and Terrestrial Megafauna Research Cluster University of the Sunshine Coast Sippy Downs Queensland Australia; ^3^ New South Wales Department of Primary Industries & Regional Development Sydney Institute of Marine Science Mosman New South Wales Australia

**Keywords:** predator avoidance, predator–prey interactions, shark‐inflicted injuries, *Sousa sahulensis*, *Tursiops aduncus*

## Abstract

Predation and its risk influence the ecology and evolution of both predator and prey species. Despite this, predatory attempts of large apex sharks on cetaceans often remain unobserved, constraining empirical assessments of their frequency and ecological significance. Shark bite scars can be used as an indirect measure to quantify predation risk on dolphins and may reveal species‐specific and spatial patterns of predator–prey interactions. Here, we analysed photographs of coastal dolphins in southeast Queensland, Australia to compare predation risk between multiple dolphin species across differing habitats. Using fresh wounds, bites were mainly attributed to tiger ( *Galeocerdo cuvier* ) and white ( *Carcharodon carcharias* ) sharks, with the peduncle being the most bitten body region across all species. Shark bite scarring differed between species: 50.3% of Australian humpback ( *Sousa sahulensis* ), 27.7% of Indo‐Pacific bottlenose ( *Tursiops aduncus* ) and 38.5% of common bottlenose ( *Tursiops truncatus* ) exhibited scars. Dolphins had more scars in sheltered waters (42.6%) compared to open waters (16.3%). Generalised linear models confirmed *S. sahulensis* were more susceptible to predation attempts, with non‐calves in sheltered waters most at risk. These findings provide a baseline for current predation risk across multiple habitats of sympatric dolphin species in Queensland, providing insight into drivers of the predator–prey interactions.

## Graphical abstract

Shark bite scars were analysed on coastal dolphins in southeast Queensland, Australia to compare the predation risk between species and habitats. Australian humpback dolphins ( *Sousa sahulensis* ) exhibited the highest bite rates and individuals found in sheltered waters had higher shark bite scar prevalence than open waters. These findings provide a baseline for shark‐dolphin predation risk in sympatric dolphin species in coastal cetacean communities.

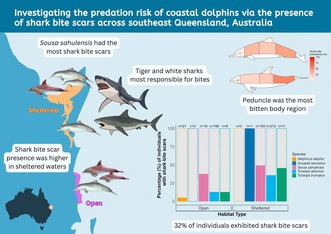

## 1 Introduction

Predation risk is a critical driver in ecology and can help shape the distribution, group size, group competition and habitat use of prey species (Fryxell et al. 2007; Lima and Dill 1990; Norris and Schilt 1988; Wirsing et al. 2008). However, depending on the probability of predator–prey interactions, the relative importance of predation risk to individuals varies between species, habitats and populations (Heithaus and Dill 2006; Labra and Niemeyer 2004). Although often considered top predators, small cetaceans are still at risk of predation from both large sharks and orcas ( *Orcinus orca* ), particularly in waters where the range of these predatory species overlaps (Heithaus 2001b; Weller 2009). Predation risk for small cetaceans varies between tropical and temperate regions; in temperate waters the risk tends to originate from orcas, whereas the risk in tropical ecosystems is typically dominated by sharks (Weller 2009). These threats have been demonstrated to influence delphinid movement, behaviour and overall habitat use (Connor and Heithaus 1996; Heithaus and Dill 2002). Despite general knowledge regarding predators, for many small cetacean populations there is currently no information on predation risk. Identification of predation risk, rate, and how it shapes dolphin movements is vital to our understanding of how it might drive cetacean behaviours and population dynamics. These drivers have the ability to influence dolphin prevalence in time and space, and are important precursors to understanding their broader ecology (Heithaus and Dill 2002).

In tropical and sub‐tropical waters, large sharks play an important role in regulating the food web, predating on sick, injured and vulnerable species, including small cetaceans (Heithaus and Dill 2002; Kiszka et al. 2015; Wirsing et al. 2008). Species such as bull ( *Carcharhinus leucas* ), tiger ( *Galeocerdo cuvier* ) and white ( *Carcharodon carcharias* ) sharks all undergo ontogenetic dietary shifts as they grow, expanding their diet from smaller fishes and crustaceans to include other elasmobranchs, turtles, and marine mammals like dugongs and delphinids (Grainger et al. 2020; Heithaus 2001a; Kim et al. 2012; Malcolm et al. 2001; Salinas‐de‐León et al. 2019; Türtscher et al. 2022). These shifts are intrinsically linked to increasing energetic requirements in relation to sexual maturity, along with physiological changes in jaw gape and tooth morphology (Ferrara et al. 2011; French et al. 2017; Goodman et al. 2022; Holmes et al. 2015). Dolphin blubber constitutes a rich food source, and therefore the hunting strategies employed by larger sharks may involve attempts at singular bites (Heithaus 2001b). A single bite would likely provide substantial energetic gain, while minimising the shark's energetic expenditure (and risk of injury) needed to exhibit a full lethal attack on a dolphin (Heithaus 2001b). In the presence of predatory sharks, dolphins have been shown to move to deeper, more open waters (Heithaus and Dill 2002; Sprogis et al. 2018), spend time in larger groups in shallower waters (Heithaus and Dill 2002), increase their speed when travelling, and undertake increases in leap frequency to avoid potential attack (Connor and Heithaus 1996). These predator–prey dynamics may shape habitat preference for where the dolphins choose to rest and forage, as habitat selection reflects trade‐offs between resource availability and increased predation risk.

Shark bites are distinguished from other markings as they are typically crescent shaped, jagged and have widely spaced tooth marks (Heithaus 2001a). Dolphins exhibit a relatively fast healing process (1–6 months) (Corkeron et al. 1987b; Orams and Deakin 1997), therefore, once the bite is fully healed, species identification and size estimates of the shark responsible are difficult to distinguish as the characteristics (i.e., tooth spacing) of the bite become harder to determine (Smith et al. 2018). There have been only a few studies where shark bite species identification has been achieved. For example, in the tropical Kimberly region of northwestern Australia, *G. cuvier* bites were the most prevalent across three coastal dolphin species (Smith et al. 2018), while in Moreton Bay, Queensland, Australia, predation attempts on dolphins were attributed to both *G. cuvier* and *C. carcharias* (Corkeron et al. 1987a). These regional differences highlight the importance in identifying which shark species prey upon dolphins to assess spatial variation in predation risk and differences in predator–prey dynamics.

One of the challenges that comes with quantifying shark predation risk is successful shark predatory events are rarely observed in the wild (e.g., Sucunza et al. (2015)). More recently, through advancements in genomic approaches, studies have been able to use environmental DNA (eDNA) metabarcoding from cloacal swabs of sharks to confirm marine mammal predation (Clark et al. 2023), an advancement on traditional stomach content analyses (Cockcroft et al. 1989; Grainger et al. 2020; Heithaus et al. 2017). However, the use of both stomach contents and eDNA analyses does not provide a complete overview of the predation risk to dolphins, as it does not account for the failed predation attempts if no food source was gained by the shark during the attempt (i.e., only teeth marks are left rather than gaining a bite of flesh) (Heithaus 2001a). These failed shark predation attempts, commonly seen on dolphins as visual bite scars and wounds, can be used to help infer predation risk and rate to dolphins (Heithaus 2001a; Heithaus and Dill 2006). In the absence of shark specimens (stomach contents or cloacal swab availability), when trying to recognise the predation risks within‐ and between‐ dolphin populations, the presence, absence, frequency and any trends of these wounds and scars can be quantified to inform predation risk (Heithaus 2001a). As such, this study uses bite wounds to serve as a proxy for predation risk which can then be used to infer how this risk might be driving dolphin population dynamics, habitat selection, and changes in both social and foraging behaviour.

It is important to note that shark bite scars document the survival of an individual following an attack (in other words a failed predation attempt). Documenting these wounds does not necessarily provide a full measure of predation pressure but does serve as a proxy for the risk of predation (Heithaus 2001a, Heithaus and Dill 2006). Inevitably, dolphins that succumb to a successful predation attempt are unaccounted for in these measures as a scar is not present to assess. Likewise, the absence of bite scars does not necessarily infer no predation risk, but likely (though not equivocally) suggests a lower risk. Therefore, though the use of shark bite scars to assess predation risk is flawed, it is a common measure used (Heithaus 2001a; Heithaus and Dill 2006; Moxley et al. 2026; Smith et al. 2018; Sprogis et al. 2018) that provides the most accurate estimates we can obtain without observation of predation events or a full understanding of predatory shark diet and movement within a region.

The frequency of shark bites has been shown to vary between dolphin species, habitats and years. For example, in a resident population of common bottlenose dolphins ( *Tursiops truncatus* ) in Sarasota Bay, Florida, shark bite frequency increased to 35.5% in 2015 (Wilkinson et al. 2017) from 22% between 1975 and 1985 (Wells 1991; Wells et al. 1987). In northwestern Australia, Smith et al. (2018) reported 72% of Australian snubfin dolphins ( *Orcaella heinsohni* ) displayed evidence of shark predation attempts, compared to 46% of Australian humpback dolphins ( *Sousa sahulensis* ) and 18% of Indo‐Pacific bottlenose dolphins ( *Tursiops aduncus* ) in the same region. The only habitat comparison of shark bite scarring on live animals was reported by Sprogis et al. (2018), noting the proportion of shark bites was higher in dolphins that resided in sheltered waters compared to those in open waters. However, Moxley et al. (2026) found globally coastal dolphins appear to experiencing predation pressure at a far lower rate than expected. These are all examples of why greater understanding of how local environmental factors, coupled with seasonal shark movements, are needed to determine the predation risk for each dolphin population. Only then can the predator–prey dynamics of a region and the subsequent influences that this may have on dolphin behaviour, ecology and habitat be better understood at both a temporal and spatial scale. However, there remains a paucity of quantified predation risk for many dolphin populations. Baseline assessments are particularly critical in densely populated coastal regions, where dolphins have cumulative pressures from both natural threats (i.e., predation) and anthropogenic pressures such as pollution, habitat loss and vessel traffic (Pirotta et al. 2013, 2015). Understanding baseline predation risk in these areas is essential for monitoring changes, as anthropogenic disturbances may alter dolphin behaviour and habitat use leading to increased susceptibility in predation risk.

In this study, we used photographic evidence to investigate the natural threat to dolphins present in south‐east Queensland (hereafter ‘SEQ’) (inclusive of the Sunshine Coast and the Great Sandy Marine Park, hereafter ‘GSMP’) via the presence of shark bite scarring. There remains a paucity of information on the natural threats of dolphins in SEQ outside Moreton Bay (Corkeron et al. 1987a; Hawkins et al. 2022; Moxley et al. 2026), and how these threats impact dolphin behaviour and ecology. Therefore, we assessed predation attempts on dolphins commonly found in SEQ ( *Delphinus delphis* , *O. heinsohni, S. sahulensis*, *T. aduncus* and *T. truncatus* ) to: (1) identify which shark species were responsible for predation attempts; (2) assess if there were differences in the distribution of bites on the body area; (3) determine if unsuccessful shark predation attempts differ by dolphin species, habitat type, age class, group size and depth; and (4) investigate if the number of bites per individual differed between species and habitat type.

## 2 Methods

### 2.1 Study Area

**FIGURE 1:**
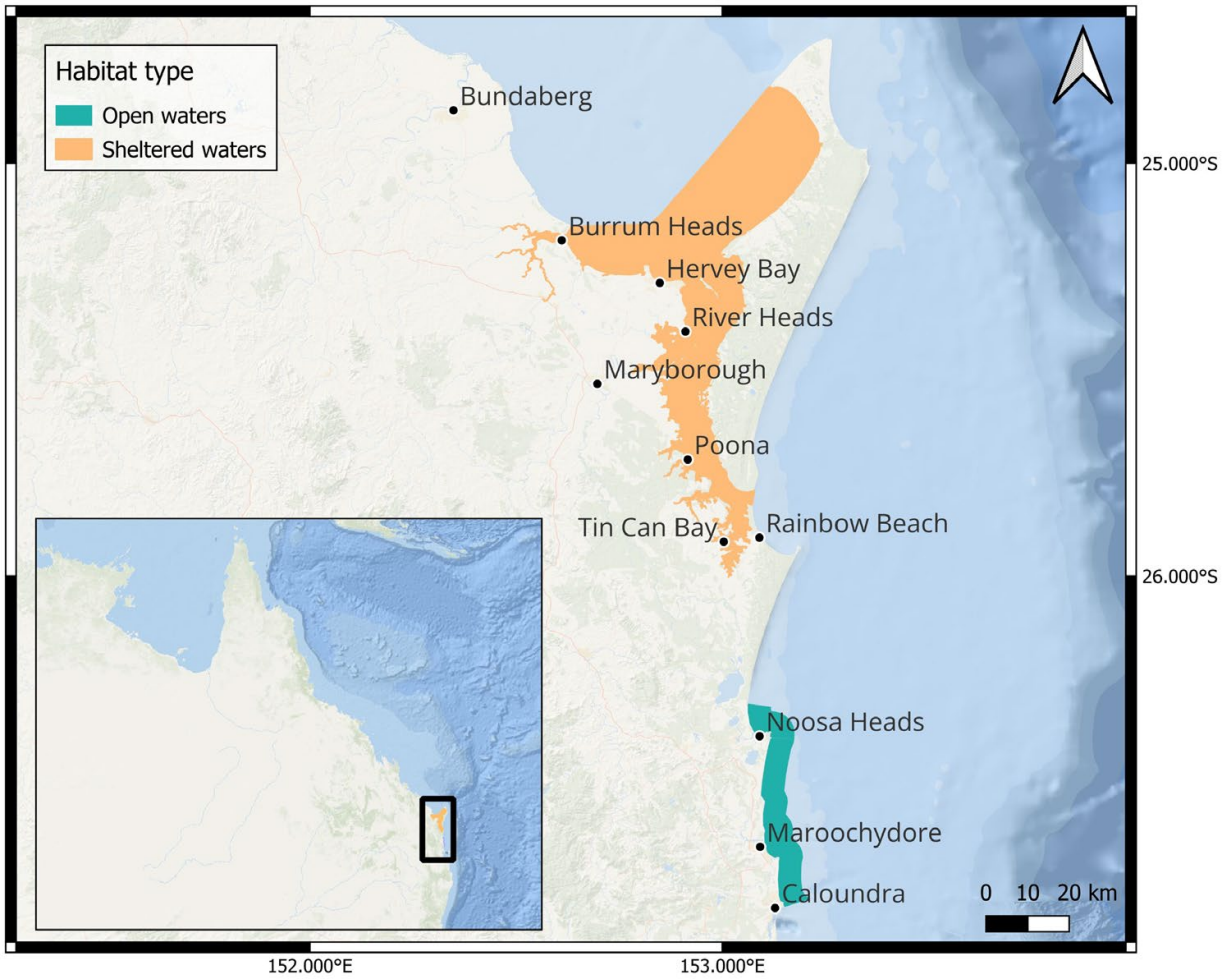
Study site location in southeast Queensland, Australia, inclusive of the ‘sheltered’ waters of the Great Sandy Marine Park (orange) and the ‘open’ coastal waters of the Sunshine Coast (teal).

Data were collected year‐round in SEQ, Australia in the GSMP (25.35° S, 152.95° E) inclusive of Hervey Bay, the Great Sandy Straits and Tin Can Inlet (Butchulla Country), and along the coastal waters of the Sunshine Coast from Noosa to Caloundra (Gubbi Gubi/Kabi Kabi Country, 26.55° S, 153.12° E) (Figure 1). The area surveyed in the marine park was approximately 2800 km^2^, whereas, to keep habitat features comparable (i.e., depth limits) the Sunshine Coast survey area was smaller, consisting of approximately 350 km^2^ of coastal (< 3 nm) and offshore waters (up to 10 nm). ‘Open’ waters hereafter consist of the ‘Sunshine Coast’ waters, comprising unprotected sandy beaches, small embayment's, rocky headlands and river access points. These waters are frequently subjected to large swell from the Pacific Ocean and feature a sloping bottom that reached a maximum depth of 31.9 m within the sampled area. Conversely, ‘sheltered’ waters of the GSMP are not subjected to this consistent swell, being protected and constrained by mainland Australia on the west, and the sand island K'gari (Fraser Island) to the east. The bay contains shallow coral reefs, intertidal wetlands, mudflats, seagrass beds, shifting sandbars, a number of estuaries and rivers flowing into the system and a mean water depth of 15 m (Gräwe et al. 2009).

Several dolphin species are predicted to occur throughout SEQ waters (Jefferson et al. 2015), however published research exists for only a fraction of these, concentrated within two marine parks: the Moreton Bay Marine Park ‘MBMP’ to the south of our study region and the GSMP (Ansmann et al. 2013; Cagnazzi et al. 2011; Corkeron 1990; Hawkins and Good 2026). Dolphin species within the MBMP are relatively well understood; with insight into species presence, habitat selection, predation risk and genetic structuring having occurred over the last 30 years (Ansmann et al. 2013; Corkeron 1990; Hawkins et al. 2022; Meager et al. 2018; Paterson and Paterson 2001; Schultz and Corkeron 1994). Comparatively, fewer studies have occurred in the GSMP (Cagnazzi et al. 2011; Hale et al. 2000; Parra et al. 2018), and these primarily focused on *S. sahulensis* with only one study assessing distribution within the Straits of the GSMP (Cagnazzi et al. 2011). Importantly, to date there is no published literature occurring for dolphins occurring in the SC. In addition, there is very limited understanding into the fine‐scale habitat use of sharks across SEQ, and those that have been conducted spang a much broader range (Lipscombe et al. 2020; Spaet et al. 2020). Seasonal shifts into Queensland waters have been noted for *C. carcharias* and *G. cuvier* (Holmes et al. 2014; Lipscombe et al. 2020; Spaet et al. 2020) whereas *C. leucas* use coastal and estuarine environments for pupping (Pirog et al. 2019). However, Queensland Shark Control Program logbook data identifies year‐round captures of all three species (Department of Primary Industries 2025), suggesting predation risk from all three species is present year‐round.

### 2.2 Data Collection

Data was collected as part of a larger project investigating dolphin connectivity in SEQ. Opportunistic, boat‐based surveys occurred between 2022 and 2025 using a 5.5 m vessel with a Yamaha 115 hp. outboard motor. Individual dolphins were photographed using a Nikon D7500 DSLR camera with a 100–400 mm telephoto zoom lens. Upon encountering a dolphin or group, the following data were collected: dolphin species, individual ID (if known), sex, location (via GPS), water depth, sea state, water temperature, group size, behavioural state and life history information (e.g., age‐class, reproductive status). Sex was determined by views of the genital area (when possible) or by consistent association with a calf (Mann et al. 2000; Smolker et al. 1992). Age‐class was defined as: calf (i.e., individual was < 2/3 of an adult size and frequently found in echelon or infant position with an adult, presumably the mother) or non‐calf (consisting of juveniles, sub‐adults and adults) (Whitehead and Mann 2000), as exact ages are not known for most of the population. A group was defined as all individuals within 10 m of another (i.e., the 10 m chain rule) (Smolker et al. 1992) as individuals beyond this distance were often exhibiting behaviours different to those within the group.

### 2.3 Data Selection

Individual dolphins were identified using standard dorsal fin photo‐identification methods including the shape, scars, nicks, wounds and pigmentation (Würsig and Würsig 1977) using photographs collected between 2022 and 2025. Photographs were graded from A (i.e., best quality) to F (i.e., not usable) as part of the photo‐identification process; photographs that were scored A‐C only were used for this study (Würsig and Jefferson 1990). Only dolphins that were able to be individually identified were included; this ensured shark bites weren't being documented multiple times when individuals were resighted on different days. For shark bite identification, photographs were inspected on the following body areas: the head, anterior, dorsal fin, mid‐flank and peduncle (as described in Scott et al. (2005), Figure 2). These body areas were chosen as they are the most readily photographed when a dolphin surfaces.

**FIGURE 2:**
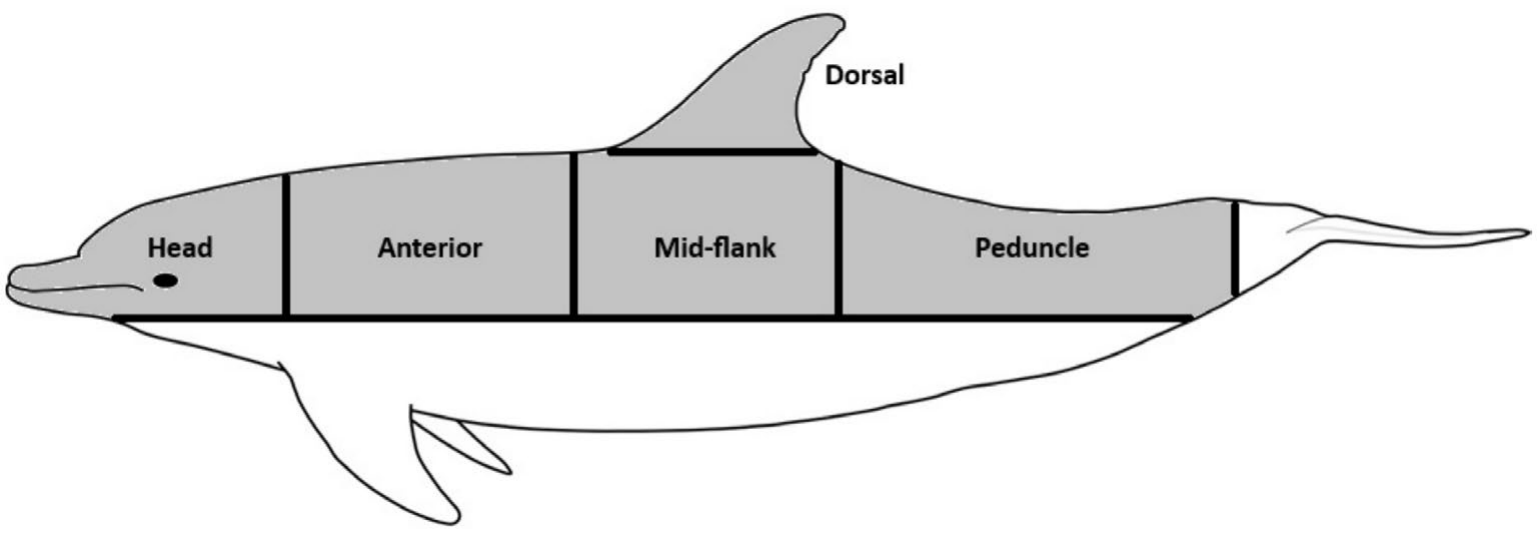
Outline of a dolphin (*Tursiops* spp.) illustrating the body observed for the presence of shark bite scars.

All individually identifiable dolphins were included in the descriptive results to allow for a broad overview between species across SEQ. However, to ensure scar presence was not biassed towards individuals with greater photographic coverage, only individuals that had been photographed on both sides were included in the final subset that was subsequently used for statistical analysis (Heithaus 2001b; Smith et al. 2018). As such, *D. delphis* (*n* = 21) and *O. heinsohni* (*n* = 1) were removed from statistical analysis due to insufficient sample sizes. *T. truncatus* could only be included in the region‐wide analysis as they also had insufficient sample size for habitat specific analysis. This resulted in a total of 720 individual dolphins for the descriptive results and 564 individuals for the final subset used in statistical analysis.

### 2.4 Shark Bite Analysis

**FIGURE 3:**
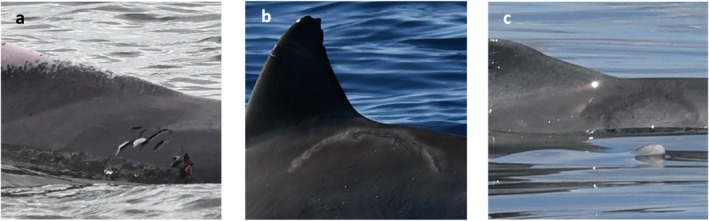
Shark bite wounds on *Sousa sahulensis* (a, c) and *Tursiops aduncus* (b) photographed in the Great Sandy Marine Park, Queensland. (a) represents an ‘Open’ (O) wound, (b) represents an ‘Intermediate’ (I) wound, (c) represents a ‘Healed’ (H) scar.

All individuals in the photo‐identification catalogue were assessed visually for evidence of shark bites. Shark bites differ from other marks attributed to dolphins or other species (e.g., bird rakes) as they are typically jagged, have a crescent shape, and are made of widely spaced tooth marks (Heithaus 2001a). Marks that are narrowly spaced or shallow tend to be from other dolphin interactions (Scott et al. 2005). Linear scars (sometimes attributable to plunging birds in a mixed‐species foraging event (Kügler and Orbach 2014)) and small notches on any body part were not included due to uncertainty of their origin. Each shark bite was assigned to the body area it mostly covered (> 50% of the scar) (‘L’ = left side, ‘D’ = dorsal side, ‘R’ = right side, ‘V’ = ventral side), and the healing classification (adapted from (Smith et al. 2018, Sprogis et al. 2018)) of the scar was assigned (open ‘O’ = wound from the last two months, including broken skin with some blubber, blood, or muscle shown; intermediate ‘I’ = wound is approximately 2–6 months old and shows some healing, white scarring will be seen; healed ‘H’ = wound is approximately more than six months old and is completely healed; Figure 3).

#### 2.4.1 Shark Bite Species Identification

Some shark bites can be identified to the species‐level based on the bite characteristics (Corkeron et al. 1987a). For example, *G. cuvier* have a wide head with teeth that are large and widely spaced, inflicting a slashing bite (Clua et al. 2023; Heithaus 2001a). Although both *C. carcharias* and *C. leucas* both have triangular serrated teeth on their upper jaws, the interdental distance ‘IDD’, (i.e., the distance between adjacent tooth tips (Lowry et al. 2009)) relative to the jaw circumference or bite width differs between the two; *C. carcharias* exhibits larger IDD compared to *C. leucas* which has overlapping upper jaw teeth (Clua and Reid 2018). Similarly, lower jaw bite imprints can be differentiated based on bite characteristics; *C. carcharias* exhibits a less ragged appearance than *C. leucas* due to the former possessing serrated triangular lower jaw cutting teeth (Hunt et al. 2026) compared to the non‐serrated tearing teeth of *C. leucas* (Goodman et al. 2022). However, difficulties in shark species identification occur when associated with old or partially visible (i.e., the mark from the lower jaw was underwater) wounds (Heithaus 2001b; Smith et al. 2018). As such, identification of the shark species responsible for bites was only attempted on the open and intermediate bites by two experienced shark biologists (VMP and BJH) looking for species‐specific distinctive features in the bite wounds.

#### 2.4.2 Data Analysis

All data were analysed in R version 4.4.3 (R Core Team 2024). To investigate if shark bite presence differed between the three most commonly sighted dolphin species ( *S. sahulensis* , *T. aduncus* and *T. truncatus* ) and habitat type, chi‐squared tests (χ^2^) with Bonferroni correction (alpha = 0.025) were used. To determine the minimum sample size required for inclusion in subsequent statistical analysis, a power analysis was conducted (one‐way ANOVA; f = 0.5, α = 0.05, power = 0.80; Cohen (1988)). The power analysis identified that only *S. sahulensis* and *T. aduncus* exceeded the minimum sample size threshold and therefore all remaining statistical analyses was run on *S. sahulensis* and *T. aduncus* . To assess if heterogeneity in sampling effort introduced detection bias, we first tested whether the sighting frequency per individual predicted shark bite scar presence using a binomial generalised linear model (GLM). Sighting frequency was not a significant predictor of scar presence (*β* = 0.063, *z* = 1.77, *p* = 0.077), suggesting differences in sampling effort did not bias bite presence. For each dolphin, only one measurement per individual used for the GLMs. Additionally, the average depth and average group size were calculated per individual based on the total number of sightings and included as explanatory variables. Binomial GLMs were then used to investigate shark bite presence, and Poisson GLMs to investigate the number of bites per individual, with dolphin species, age class, habitat type, average group size, average depth and their interactions included as possible explanatory variables. Sex was excluded due to inconsistencies in being able to confidently and consistently identify sex without error. The dredge function (MuMIn package; Bartoń (2022)) was used for both the shark bite presence and the shark bite number models to generate model selection tables with all possible combinations (subsets) of fixed terms for each global model and to determine the optimal model by selecting the model with the lowest AICc. Models within ΔAICc < 2 were considered competitive, the final model was chosen as the one with the smallest AICc (Table S1). Finally, to investigate species differences in where bites occurred on the body area; body specific heat maps were created for the most prevalent species ( *S. sahulensis* and *T. aduncus* ), and Fisher's Exact tests were run to statistically test for body area differences.

## 3 Results

Between 2022 and 2025, 166 survey days were completed, totalling 599 dolphin surveys across the region. This resulted in six *D. delphis* sightings, one *O. heinsohni* sighting, 173 *S. sahulensis* sightings, 382 *T. aduncus* sightings, 21 *T. truncatus* sightings and 16 sightings where species identification could not be determined due to brevity in sighting/loss of individuals. From these surveys, a total of 720 individually identifiable dolphins were catalogued: 207 *S. sahulensis*, 472 *T. aduncus* , 19 *T. truncatus* , 21 *D. delphis*, and one *O. heinsohni* . Individuals were sighted an average of 2.81 ± 2.16 times (range 1–13) and group sizes ranged from one to ca. 50 individuals (average 10.96 ± 8.56).

**FIGURE 4:**
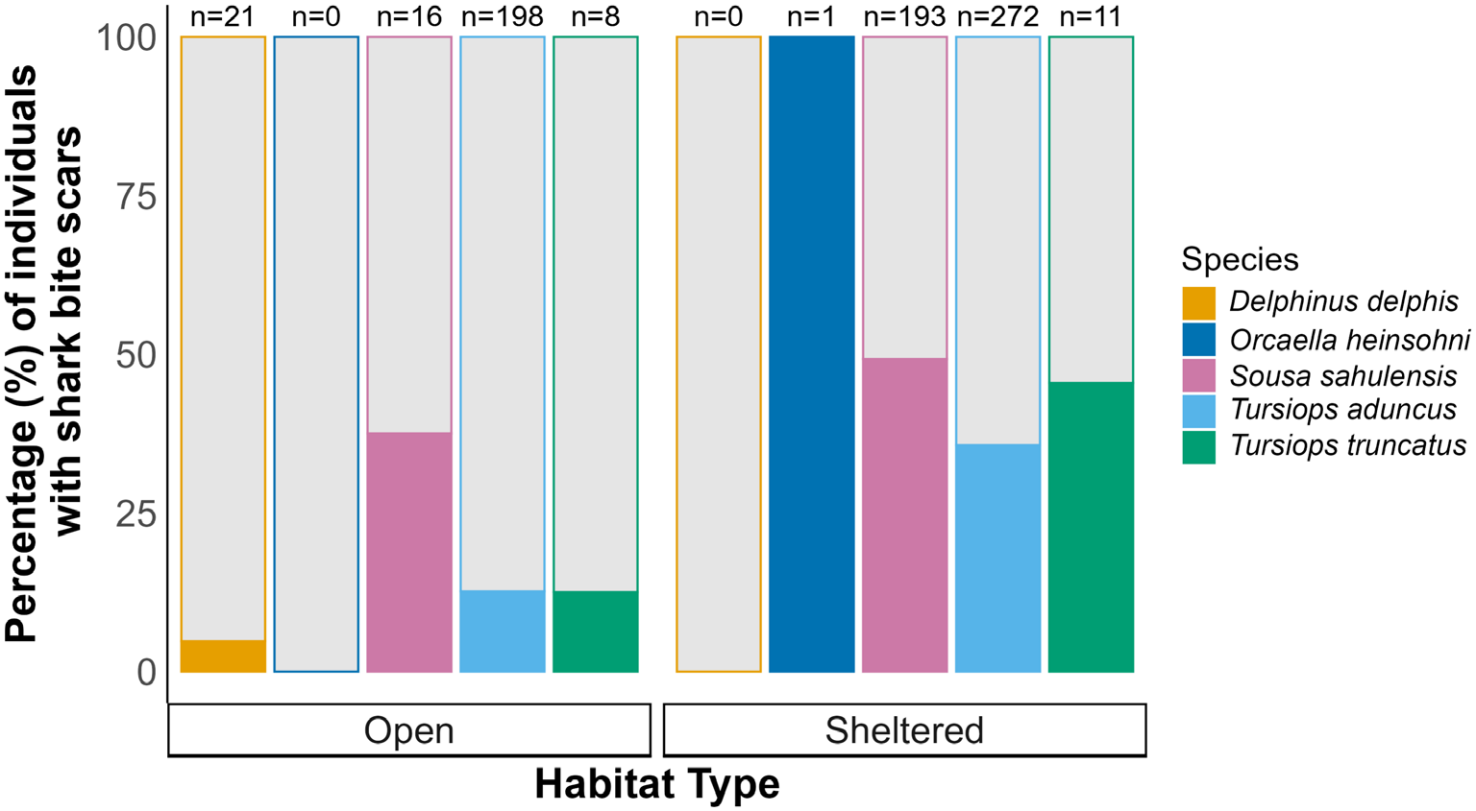
Percentage of individual dolphins exhibiting shark bite scarring (*Delphinus delphis*, *Orcaella heinsohni* , *Sousa sahulensis*, *Tursiops aduncus* and *Tursiops truncatus* ) from the Great Sandy Marine Park (sheltered) and the Sunshine Coast (open), Queensland, Australia. The grey area represents the percentage of each species that did not exhibit shark bite scarring, and ‘*n*’ represents the number of individuals of that species in the study.

A total of 338 shark bites were identified on 231 of the 720 dolphins, resulting in 32% of the SEQ dolphin population surviving predation attempts. The presence of shark bite scars differed between species ( *D. delphis* (4.8%, *n* = 21), *O. heinsohni* (100%, *n* = 1), *S. sahulensis* (48.3%, *n* = 209), *T. aduncus* (26.0%, *n* = 470), *T. truncatus* (31.6%, *n* = 19)). The presence of bites was higher for all species in sheltered compared to open water habitats (Figure 4). These bites were categorised as Open (*n* = 22, 6.5%), Intermediate (*n* = 46, 13.6%) and Healed (*n* = 270, 79.9%) wounds (Table 1). The peduncle was the most bitten body region (*n* = 171, 50.6%), followed by anterior (*n* = 78, 23.1%), mid‐flank (*n* = 68, 20.1%), dorsal (*n* = 15, 4.4%), and the head (*n* = 6, 1.8%) (Table 1).

**TABLE 1:** Observed count of shark bite scars in southeast Queensland, split by body region and associated healing stage that were observed between 2022 and 2025 on *Delphinus delphis, Orcaella heinsohni, Sousa sahulensis, Tursiops aduncus* and *Tursiops truncatus* .

Scar classification		*D. delphis* (*n* = 1)	*O. heinsohni (n* = 1*)*	*S. sahulensis* (*n* = 173)	*T. aduncus* (*n* = 154)	*T. truncatus* (*n* = 9)
Body region	Anterior	0	1 (100%)	52 (30.1%)	23 (14.9%)	2 (22.2%)
	Dorsal	0	0	6 (3.5%)	8 (5.2%)	0
	Head	0	0	3 (1.3%)	2 (1.3%)	1 (11.1%)
	Mid‐flank	0	0	36 (20.8%)	31 (20.1%)	1 (11.1%)
	Peduncle	1 (100%)	0	76 (43.9%)	90 (58.4%)	5 (55.6%)
Healing stage	Open	0	0	17 (9.8%)	4 (2.6%)	1 (11.1%)
	Intermediate	0	0	22 (12.7%)	23 (14.9%)	1 (11.1%)
	Healed	1 (100%)	1 (100%)	134 (77.5%)	127 (82.5%)	7 (77.8%)

### 3.1 Shark Species Identification

Shark species identification was possible for 15 of 22 open and intermediate bites based on tooth spacing and bite characteristics. Four species/genera of sharks were characterised as the suspected predators. The most common species responsible for shark bites were *G. cuvier* and *C. carcharias* (*n* = 6, Table 2). Bites from *C. leucas* were only attributed to *S. sahulensis* (Table 2).

**TABLE 2:** Identified shark species responsible for inflicting bites on a subset of open and intermediate wounds (*n* = 15) found on two species of coastal dolphins in south‐east Queensland.

Suspected shark species	*Sousa sahulensis*	*Tursiops aduncus*
Tiger shark ( *Galeocerdo cuvier* )	4	2
White shark ( *Carcharodon carcharias* )	1	5
Bull shark ( *Carcharhinus leucas* )	2	0
Carcharhinid sp.	1	0
Total identified	**8**	**7**

### 3.2 Distribution of Shark Bites by Body Area

**FIGURE 5:**
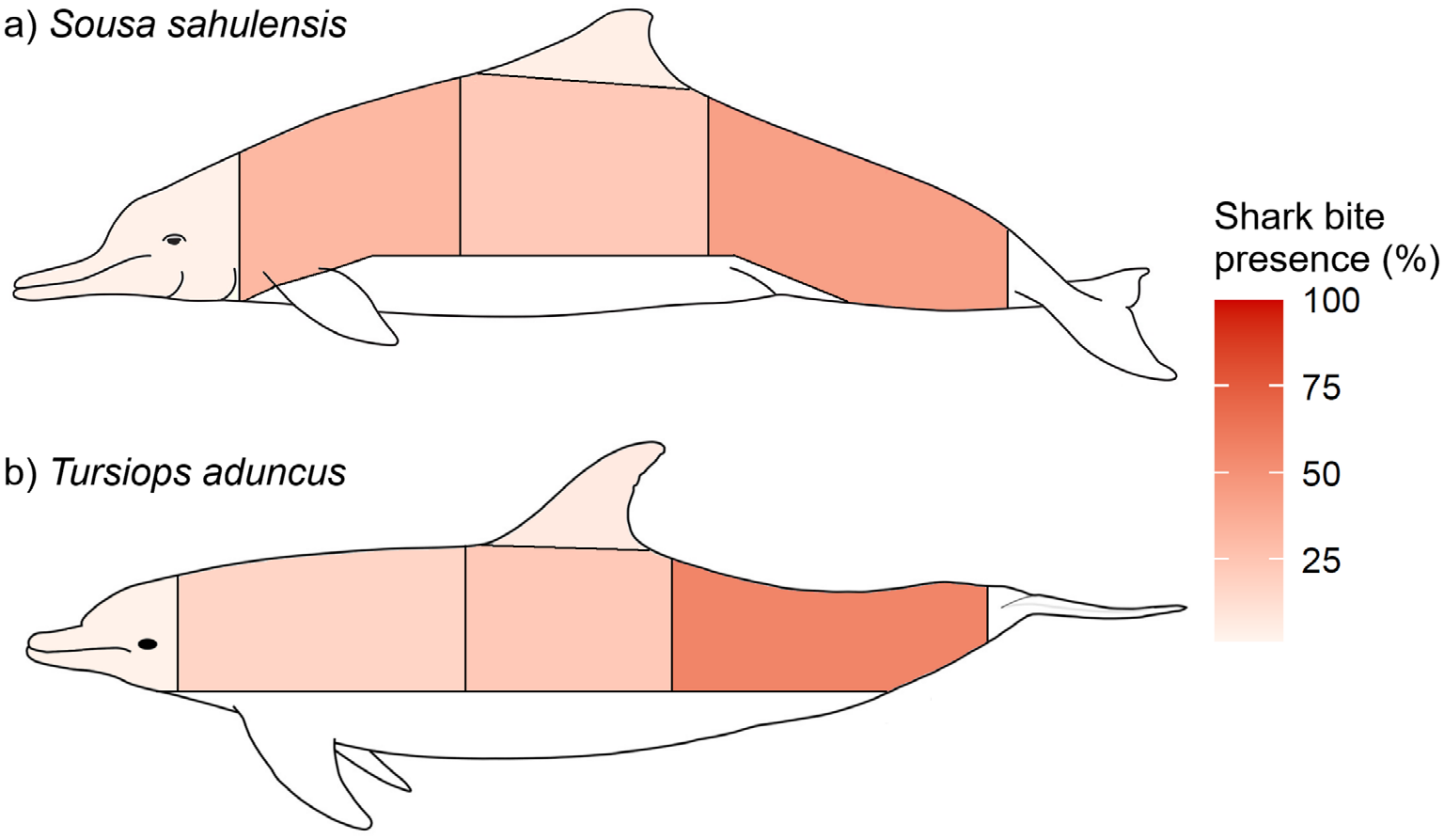
Heatmap of the distribution of shark bite scars between 2022 and 2025 across different body regions for *Sousa sahulensis* (a) and *Tursiops aduncus* (b) in southeast Queensland. Darker shading indicates higher bite occurrence.

There was no significant difference between bites on the left and right sides of individuals exhibiting shark‐bite scarring ( *S. sahulensis* : χ^2^ = 0.78, df = 1, *p* = 0.38*; T. aduncus*: χ^2^ = 1.05, df = 1, *p* = 0.31), therefore, sides were not included in analysis. The distribution of scarring across the body region of *S. sahulensis* was not random (*p* < 0.001) with most shark bites occurring in the peduncle region (42.5%), followed by the anterior (30.6%), mid‐flank (21.9%), dorsal (3.1%) and head (1.9%) (Figure 5a). The distribution of scarring across the body region of *T. aduncus* was also not random (*p* < 0.001) with most shark bites occurring on the peduncle region (55.6%), followed by the mid‐flank (21.8%), anterior (15.8%), dorsal (5.3%) and head (1.5%) (Figure 5b).

#### 3.2.1 Variation in Shark Bite Presence Between Species and Habitat Type

After filtering for the subset of individuals that included photographs of both sides, the presence of shark bite scarring significantly differed between species for the SEQ region ( *S. sahulensis* = 50.3%, *n* = 179; *T. aduncus* = 27.7%, *n* = 372; *T. truncatus* = 38.5%, *n =* 13; χ^2^ = 27.1, df = 2, *p* < 0.001). Overall, *S. sahulensis* were significantly more likely to exhibit shark bite scars than *T. aduncus* (χ^2^ = 26.1, df = 1, *p* < 0.001) yet there was no difference in bite presence between *T. truncatus* and other species ( *S. sahulensis* : χ^2^ = 0.29, df = 1, *p* = 0.59; *T. aduncus* : χ^2^ = 0.29, df = 1, *p* = 0.59).

**FIGURE 6:**
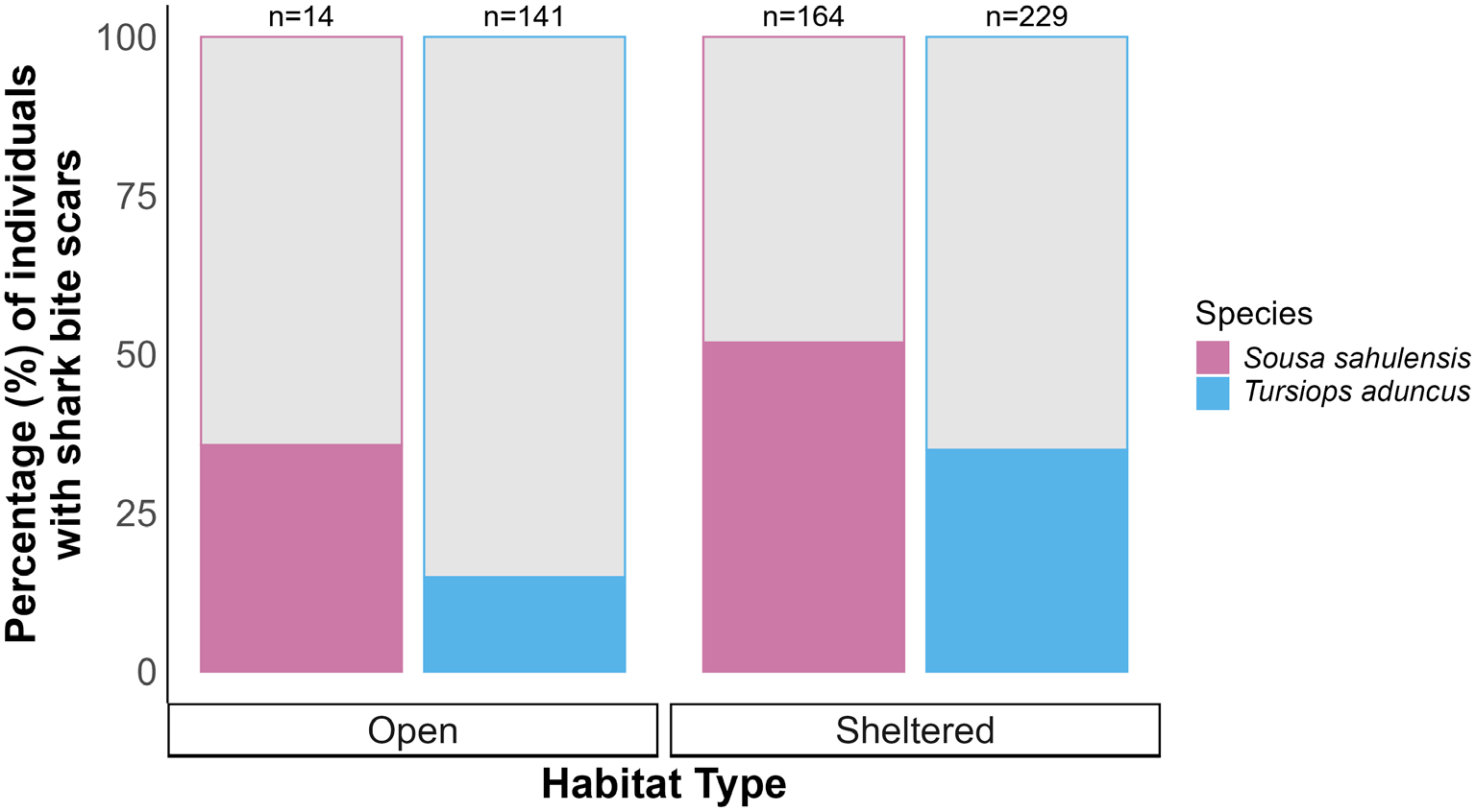
Presence of shark bite scarring in *Sousa sahulensis* and *Tursiops aduncus* for which photographs exist of both sides of each individual within the Great Sandy Marine Park (sheltered) and the Sunshine Coast (open), Queensland, Australia. The grey area represents the percentage of each species that did not exhibit shark bite scarring, and ‘n’ represents the number of individuals of that species in the study.

Dolphins found in open water habitats were significantly less likely to exhibit shark bite scars than those found in sheltered habitats (open = 16.3%, *n* = 160; sheltered = 42.6%, *n* = 402; χ^2^ = 33.7, df = 1, *p* < 0.001). However, the results differed when exploring the species differences within each habitat type; *S. sahulensis* were significantly more likely to have shark bite scars than *T. aduncus* in sheltered water environments ( *S. sahulensis* = 51.5%, *T. aduncus* *=* 35.5%: χ^2^ = 9.48, df = 1, *p* = 0.002), but no difference was seen between species in open waters ( *S. sahulensis* *=* 35.7%, *T. aduncus =* 14.9%: χ^2^ = 2.60, df = 1, *p* = 0.11) (Figure 6).

#### 3.2.2 Factors Influencing Shark Bite Presence

**FIGURE 7:**
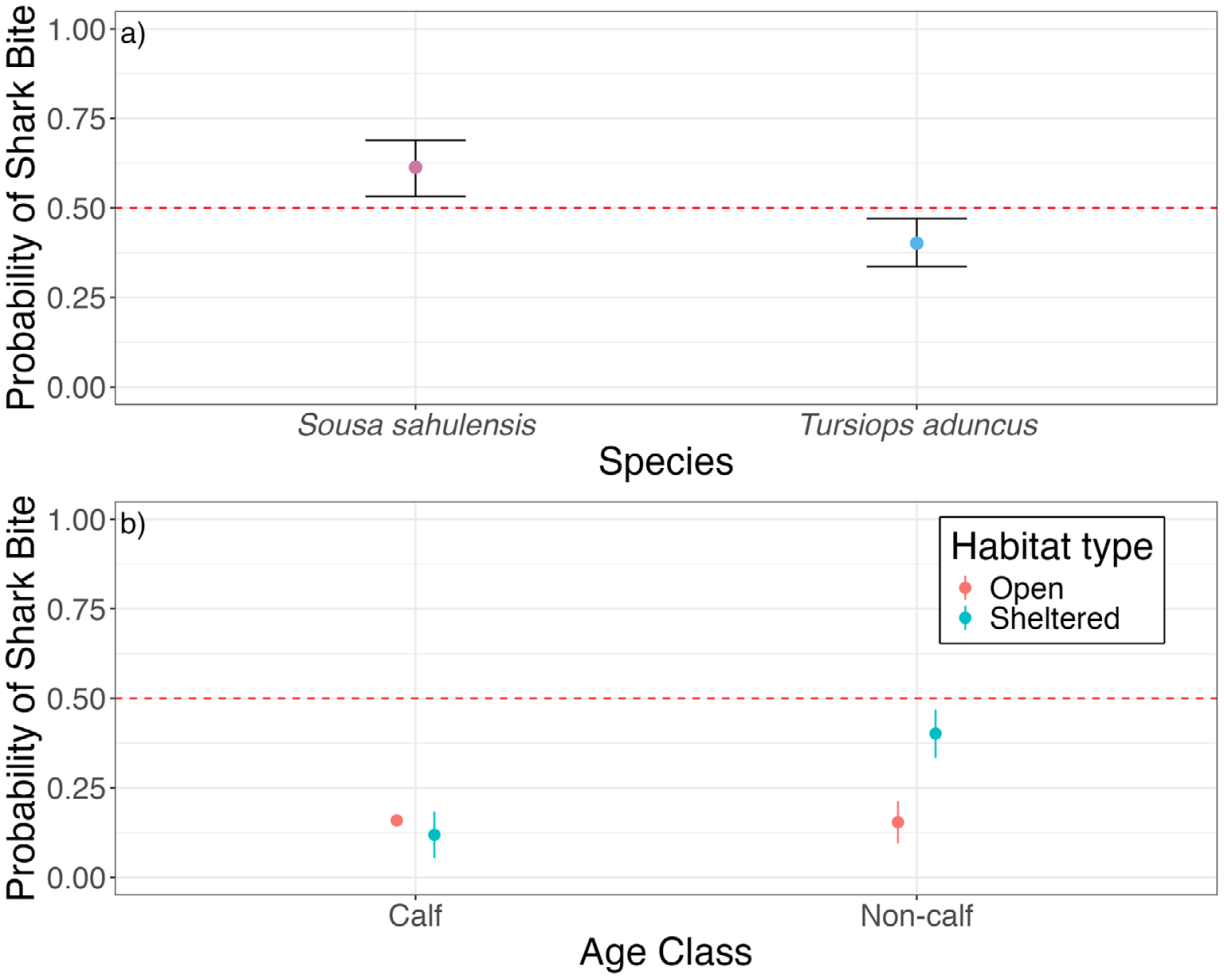
Significant drivers of the presence of a shark bite in dolphins in southeast Queensland, Australia for (a) dolphin species and (b) the interaction of age class and habitat type. Error bars represent 95% confidence intervals, and the red dashed line represents the equal probability of a dolphin exhibiting a shark bite. There is no confidence interval shown for calves in open waters due to only one individual photographed exhibiting a bite scar.

The top‐ranked GLM indicated three variables, species, habitat, and age had an influence on shark bite presence; however, neither average group size nor average depth were significant (model selection is listed in Table S2). Species differences showed that *S. sahulensis* were significantly more likely to exhibit shark bite scarring than *T. aduncus* (Figure 7a). The interaction between age class and habitat was also significant, demonstrating that non‐calves in sheltered habitats were more likely to exhibit shark bites than non‐calves in open habitats or calves in either habitat type (Figure 7b, Table 3).

**TABLE 3:** Binomial generalised linear model (GLM) results of the top‐ranked model (predictors: Age class, habitat, species) for shark bite presence on dolphin species in southeast Queensland. The reference is *S. sahulensis* calves in open water habitats. Significance denoted in bold.

Model	Predictor	Estimate (β)	SE	*z*‐value	*p*‐value
Bite presence	Intercept	−0.81	0.66	−1.21	0.225
	Age Class (Non‐calf)	−0.04	0.68	−0.06	0.956
	Habitat Type (Sheltered)	−0.34	0.71	−0.48	0.633
	Species ( *T. aduncus* )	−0.86	0.21	−4.14	**< 0.001**
	Age Class (Non‐calf) × Habitat Type (Sheltered)	1.64	0.75	2.19	**< 0.05**

### 3.3 Frequency of Shark Bite Scarring Between Species

GLM results for number of bites showed *S. sahulensis* had a significantly higher mean number of bites per bitten individual (1.71 ± 0.952 bites (*n* = 101)), compared to *T. aduncus* (1.26 ± 0.586 bites (*n* = 122)) (Figure S1, Table S1). No other predictors were found to influence the number of bites per individual (Table S2).

## 4 Discussion

The ecological importance of predator–prey relationships is key in shaping marine communities; however, predation risk is often overlooked for many dolphin populations. This study addresses this gap for SEQ, providing the first sympatric dolphin species predation risk assessment across multiple habitats in the region, and only the second study globally to examine this risk across a range of habitats (i.e., open, coastal waters and sheltered embayments). All five dolphin species examined experienced shark predation attempts, indicating shark predation is a threat across all dolphin species in the region. Through analysis of fresh bites, *G. cuvier* and *C. carcharias* were identified as the main predators responsible for bite wounds on dolphins in the region, primarily targeting the peduncle. However, as the scars identified only represent the ‘failed’ predation attempts (Heithaus 2001b; Smith et al. 2018), the shark predation risk provided here must be considered a minimum estimate of predation in the region.

### 4.1 Distribution of Shark Bite Scarring

The majority of shark bite scarring was present on the peduncle for both *S. sahulensis* and *T. aduncus*, followed by the anterior (for *S. sahulensis* ) or mid‐flank ( *T. aduncus* ). Comparatively, the head and dorsal fin regions had the lowest amount of scarring present. Although this photo‐identification method does not account for bites to the ventral side of the dolphins, scarring patterns on the dorsal side of the individual observed here are consistent with other studies that suggest dolphins turn their dorsal side towards the shark to evade an attack and protect their vital organs located closer to their ventral side (Cockcroft et al. 1989; Heithaus 2001b; Smith et al. 2018). Consequently, bites to the ventral side or head of the dolphin are likely to be fatal (Heithaus 2001b), explaining the absence of bites in these body regions as individuals are unlikely to survive these predation events. Additionally, sharks are known ambush predators that likely commence predation attempts from below and out of the field of visual or acoustic detection (Geraci et al. 1978), particularly while the dolphin is surfacing to breathe (Heithaus and Dill 2002; Smith et al. 2018). It is important to acknowledge the lower number of bites on the head and absence of bites on the ventral region could be related to a lack of photographic coverage rather than a true absence of wounds, and for *S. sahulensis* the mid‐flank region often did not fully break the surface of the water, further limiting detection. We therefore caution that some dolphin body regions are underrepresented; these records of unsuccessful predation attempts should be considered as minimum estimates of shark predation frequencies on dolphins in SEQ waters.

### 4.2 Shark and Dolphin Habitat Overlap

Two large shark species, *G. cuvier* and *C. carcharias* were identified as the primary predatory species impacting dolphins in SEQ (based on photographs of open and intermediate bites). Although direct predatory observations on dolphins by these species are scarce, dietary analysis of stomach contents has revealed that ontogenetic shifts to include mammal prey occur at approximately 2.5 m total length (TL) for *C. carcharias* (Clark et al. 2023; Grainger et al. 2020; Kim et al. 2012). Similarly, an ontogenetic shift in prey to include marine mammals has been recorded for *G. cuvier* in both Hawaii (Lowe et al. 1996) and South Africa (Dicken et al. 2017). The latter study highlights the importance of small odontocetes, particularly *T. aduncus* and *D. delphis* , in the diets of sharks < 220 cm precaudal length (i.e., 440 cm TL see Dicken et al. (2017)). To enable mammal predation, it has been suggested that the movements of these sharks may shift to habitats where dolphin densities are higher (Bruce 1992).

Habitat use by *G. cuvier* and *C. carcharias* spatially and temporally overlap with dolphin populations at multiple life stages (Bruce et al. 2019; Lipscombe et al. 2020). These shark species generally occupy coastal and offshore tropical and warm temperate waters, but use of large embayments like the GSMP are also common. Both species also have wide‐ranging transcontinental movements detected through satellite tracking, indicting large home ranges (Holmes et al. 2014; Lipscombe et al. 2020; Spaet et al. 2020). Seasonality and prey availability are thought to drive these large‐scale movements of sharks (Andrzejaczek et al. 2025; Fitzpatrick et al. 2012), resulting in an overlap in habitat use with inshore dolphin populations. Spatiotemporally, the number of these sharks moving through SEQ will fluctuate daily, along with other environmental drivers that determine movement behaviour (e.g., water temperature, current strength, moon phase, other prey movement, etc.). Year‐round captures of both species in SEQ have been recorded for several years in the Queensland Shark Control Program (QSCP) logbooks (Department of Primary Industries 2025). Gear associated with the QSCP (i.e., nets and drumlines) are deployed in open coastal areas throughout SEQ, some of which also abut major river systems and the GSMP.

While open water predation risk may remain relatively constant throughout the year, seasonal fluctuations in large shark presence in sheltered estuarine waters may also cause temporal increases in predation risk. For example, the return to brackish and freshwater rivers to pup in the summer months by *C. leucas* is part of their life history strategy (Pirog et al. 2019), and increases the overlap potential with any inshore dolphin populations present. Stomach contents analysis confirm dolphin presence in *C. leucas* diets, although, dolphins only seem to represent a small proportion of their diet, with elasmobranchs and teleost's being the most common prey (Cliff and Dudley 1991; Tinhan and Wells 2021). The dietary difference might be attributed to the smaller size of adult *C. leucas* (Tillett et al. 2011) compared to larger *G. cuvier* and *C. carcharias* (Burgess et al. 2014; Meyer et al. 2014), potentially limiting their ability or need to prey on dolphins. Consequently, despite extensive spatial overlap with dolphins, only two bites in this study were attributed to *C. leucas* , and both occurred on *S. sahulensis* . This suggests although dolphins may not be preferential prey for *C. leucas* , *S. sahulensis* are experiencing greater cumulative predation risk from large sharks due to their habitat overlap with all three predating species, potentially explaining their higher bite scar prevalence observed overall.

### 4.3 Habitat's Influence on Shark Bite Prevalence

Dolphins in sheltered waters exhibited higher shark bite scarring rates than those in open waters, indicating elevated predation risk in sheltered habitats. This pattern likely reflects a combination of factors that concentrate predation risk in the shallow, sheltered habitat. The dolphins and all three of the predatory shark species identified are known to utilise embayment habitats due to the abundance of prey (Heithaus 2001a; Heithaus and Dill 2006; Meager et al. 2018), increasing the potential predator–prey encounters. Further, the dolphin's acoustic detectability is reduced in the turbid, shallow waters, increasing the success rate of shark predatory attempts in such habitats (Ebert 1991; Heithaus 2001a; Heithaus and Dill 2002). Sheltered waters surrounded by land are known to constrain the possible number of escape routes and decrease manoeuvrability of dolphins during predatory attempts from sharks compared to open waters where individuals have greater opportunity to evade the attack (Heithaus and Dill 2002). Lastly, although not a significant predictor, dolphin group sizes observed here in sheltered waters were often smaller than in open waters (Hume, pers. obvs.) which may be one of the contributing factors for the increase in predation attempts. Throughout the animal kingdom, the drivers of group size formation are complex, however, many social species that form larger groups often result in increased collective vigilance and predator detection success (Beauchamp 2003; Cresswell and Quinn 2011; Roberts 1996). This pattern has been observed with group sizes in Shark Bay, WA, where *T. aduncus* formed larger groups in shallow habitats, where *G. cuvier* shark predation risk is known to be elevated (Heithaus and Dill 2002). The slight reduction in group size in our sheltered habitat may therefore reduce the dolphin's ability to successfully detect predators, contributing to the higher shark bite rates recorded, however further research is needed to confirm this relationship.

### 4.4 Interspecific Differences in Predation Risk

Our findings suggest *S. sahulensis* face greater predation risk than *T. aduncus* populations in SEQ. Although both species are coastal dolphins with relatively sympatric habitat selection (Chilvers et al. 2005; Parra 2006), interspecific differences in dolphin behaviour and spatio‐temporal use may be driving this disparity in predation risk. *S. sahulensis* more often utilise shallower, estuarine habitats than *T. aduncus* (Jefferson and Rosenbaum 2014), exposing them to increased *C. leucas* interactions. This increase in interactions could be occurring from sub‐adult *C. leucas* still residing in the area that are learning to forage on larger prey (Heupel et al. 2007; Smoothey et al. 2019). The ontogenetic shift to targeting larger prey items like marine mammals typically occurs at approximately 2 m in length, coinciding with increased jaw gape, tooth strength, and energetic requirements (Goodman et al. 2022; Werry et al. 2011). This size‐related ontogenetic shift may explain why *C. leucas* bites appear on *S. sahulensis* . We propose that younger *C. leucas* learning to forage on larger prey will result in a higher number of unsuccessful predation attempts on these dolphins, resulting in more delphinids surviving (i.e., non‐lethal bites) and presenting as shark bite scars. Additionally, these unsuccessful predation attempts also quickly form learned behaviours by young dolphins to develop strategies to avoid sharks, like choice of habitat selection and larger group sizes (Heithaus 2001a, Heithaus and Dill 2002).

Predator avoidance behaviour by dolphins has been reported worldwide, including in Western Australia, where *T. aduncus* were found to shift their foraging activities to deeper waters in response to increases in shark numbers (Heithaus and Dill 2006). In South Africa, it has been postulated that *T. aduncus* avoid turbid waters as a potential predator avoidance behaviour (Cockcroft et al. 1989), whilst the higher level of predation on Indian Ocean humpback dolphins ( *Sousa plumbea* ) was attributed to their propensity to inhabit turbid inshore waters where predator detection may be inhibited (Cockcroft 1991). Such behavioural responses reflect how predation risk can shape habitat selection and movement patterns independently of direct predation events, as prey species consistently trade foraging opportunities for a reduction in exposure to predators (Lima and Dill 1990). Even in the absence of direct or infrequent predation, continued vigilance by prey species has been documented across many taxa (Favreau et al. 2010; Le Saout et al. 2015). For example, deer populations in Hawaii that had been isolated from predators for over 60 years still spent more than 10% of their time vigilant (Le Saout et al. 2015). Consequently, in long‐lived species like dolphins, even a low risk of predation may produce lasting antipredator behaviours, influencing overall health and fitness (Lima 1998). As such, the elevated predation attempts in sheltered waters documented in our study suggest predation risk may therefore be influencing long‐term dolphin behaviour and habitat selection beyond what the direct bite scar prevalence alone implies.

Behavioural differences between *S. sahulensis* and *T. aduncus* likely also influence the predation risk on these sympatric species. Differences in foraging strategies of these two species have been reported (Hawkins et al. 2022; Syme et al. 2023). *T. aduncus* has been observed surface foraging on epipelagic prey (e.g., bluefin trevally ( *Caranx melampygus* ) and hound needlefish ( *Tylosurus crocodilus* )) (Kiszka et al. 2014). In contrast, *S. sahulensis* has been recorded predominantly foraging on demersal prey in shallower, benthic habitats (Syme et al. 2023). This difference in foraging strategies may be influencing *S. sahulensis* to spend more time in shallower habitats where they are more vulnerable to predation attempts due to their reduced visual ability for predator detection while focusing on catching their prey. This phenomenon aligns with similar spatial patterns observed by Nicholls et al. (2023) in nearshore habitats of north Queensland, where shark bite presence increased with proximity to coast, suggesting an increase in prey abundance in nearshore habitats. In our study, *S. sahulensis* demonstrated spatial preference for slightly more estuarine waters than *T. aduncus*, which were never seen in the central and southern turbid waters of the GSMP (Hume, pers. obvs), likely driven by prey distribution (Parra 2006; Syme et al. 2023). This species‐specific microhabitat selection influences *S. sahulensis* to reside in closer proximity to the coast, potentially exposing them to increased predation risk, which is consistent with the predation‐coastal pattern previously found in Queensland waters north of our study region (Nicholls et al. 2023).

While the presence of multiple bite scars per individual suggests shark predation attempts are common, the insight that *S. sahulensis* had both a higher mean number of bites and a higher scar presence may also be reflecting species variation in shark prey preference. As generalist predators, *G. cuvier* opportunistically consume a wide range of prey including sea turtles, seabirds, pelagic fish and marine mammals when they are available (Fitzpatrick et al. 2012; Heithaus and Dill 2002), and *C. carcharias* ontogenetically shift to consume larger cetaceans and pinnipeds as they mature (Clark et al. 2023; Long and Jones 1996). Despite this dietary flexibility, the higher scar rates on *S. sahulensis* are similar to those reported in other populations in Western Australia (WA) ( *S. sahulensis* = 46.0%, *T. aduncus* = 16.9%–18.0% (Smith et al. 2018; Sprogis et al. 2018)) and Moreton Bay, Australia ( *S. sahulensis* = 48.8%, *T. aduncus* = 29.0% (Hawkins et al. 2022)), suggesting a fundamental species‐level difference in their ecology, behaviour and susceptibility to predation attempts, rather than a site‐specific effect. The consistency of this interspecific pattern across geographically distinct populations links to intrinsic differences between the two species, such as body morphology, group size, habitat fidelity, foraging differences or anti‐predator behaviours, as the potential drivers of the differential shark scar rates.

### 4.5 Age Class Differences in Shark Bite Scarring

Age class influenced bite scar rate of dolphins, as expected; juveniles and adults (i.e., non‐calves) had more bites than calves. Non‐lethal shark bite scars are expected to accumulate over time as individuals have a higher likelihood of encountering more sharks through cumulative exposure (Heithaus 2001b; Smith et al. 2018). Consequently, a large shark predatory attempt would likely be more successful on a smaller dolphin (i.e., a calf) and therefore likely fatal, potentially skewing patterns towards a higher prevalence of non‐lethal bite marks on older individuals (Heithaus 2001b; Wilkinson et al. 2017). However, as exact ages could not be determined for most of the dolphins in this study, future studies should aim to quantify age‐related patterns in shark bite scar accumulation in the region.

## 5 Conclusion

Predator–prey relationships are dynamic and intricate, with no single factor being able to explain the relationship between two species. The results of this study offer an insight into the predation risk of the coastal dolphins in SEQ and the species, habitat, and demographic information that can influence predator–prey interactions. This study can serve as a baseline for dolphin‐shark predator–prey interactions across differing coastal habitats in the region. However, ongoing efforts to disentangle how predators and prey interact are critical as predation risk influences the ecology and evolution of both predator and prey, as well as broader ecosystem dynamics. This is particularly important in one of Queensland's most densely populated regions, where establishing baseline knowledge is key prior to investigating potential anthropogenic impacts on marine species.

We acknowledge the use of shark bite scars to infer predation risk does not necessarily account for the complete ‘risk’ to the individuals; some predation attempts either result in the death of the individual and therefore not accounted for as ‘risk’ or, attempts would be unsuccessful leaving no scars. Collectively, the majority of published literature on dolphin predation risk only offers insight into dolphin ecology, limiting our full understanding of predation dynamics. To better capture the overall risk of predation, moving forward we need to integrate both predator and prey dynamics, including perspectives from both the prey (e.g., failed predation evidence, movement ecology, behaviour) and predator (e.g., shark movement/tracking studies, dietary studies). Integrating both predator and prey dynamics together (instead of a single species approach) will allow for better insights into predation and ecosystem dynamics. Additionally, long‐term research should focus on intrinsic and extrinsic drivers of dolphin‐shark interactions (e.g., shark prey availability, dolphin foraging strategies, temporal variation in shark abundance, and seasonality), facilitate understanding of the characteristics of predation risk on dolphins. Together a more holistic approach to predation risk will yield a better understanding of the dynamics of both the predator and the prey across time.

These findings highlight species‐specific differences in predation risk amongst sympatric dolphin species, suggesting habitat preferences, morphological characteristics, and behavioural strategies may influence an individual's vulnerability in shark predation risk.

## Author Contributions

**Georgina V. Hume:** data curation (lead), formal analysis (lead), funding acquisition (equal), investigation (equal), methodology (equal), project administration (equal), resources (equal), software (equal), validation (equal), visualization (equal), writing – original draft (lead). **Alexis L. Levengood:** conceptualization (equal), data curation (equal), funding acquisition (equal), investigation (equal), methodology (equal), project administration (equal), resources (equal), supervision (equal), validation (equal), visualization (equal), writing – review and editing (equal). **Gemma L. Webster:** investigation (equal), writing – review and editing (equal). **Kathy A. Townsend:** funding acquisition (equal), supervision (equal), writing – review and editing (equal). **Victor M. Peddemors:** investigation (equal), writing – review and editing (equal). **Bonnie J. Holmes:** conceptualization (equal), funding acquisition (equal), investigation (equal), methodology (equal), project administration (equal), resources (equal), supervision (lead), validation (equal), writing – review and editing (equal).

## Funding

This work was supported by University of the Sunshine Coast, Holsworth Wildlife Research Endowment & Ecological Society of Australia, Winifred Violet Scott Charitable Trust, Royal Zoological Society of New South Wales, Ethel Mary Read Research Grant and the CID Foundation.

## Conflicts of Interest

The authors declare no conflicts of interest.

## Supporting information


**Figure S1:** Mean number of shark bites per individual observed on bitten *Sousa sahulensis* and *Tursiops aduncus* in southeast Queensland. Red dotted line denotes the mean number across all species, error bars are shown.

**Table S1:** Model selection by AICc, ΔAICc (for models < 2), AICc weights and the log‐likelihood of the top models for shark bite presence and the number of bites for dolphin species in southeast Queensland, for only individuals that were photographed on both sides.
**Table S2:** Poisson generalised linear model (GLM) results of the top‐ranked model (predictor: species) of the number of shark bites per individual on dolphins in southeast Queensland. The reference species is *S. sahulensis* . Significance denoted in bold.

## Data Availability

Data supporting the result of this study will be archived on DRYAD (https://doi/org/10.5061/dryad.76hdr7t9t).
