# Supplementary figures and images for: Investigating the Predation Risk of Coastal Dolphins via the Presence of Shark Bite Scars Across Southeast Queensland, Australia
Source: Ecol Evol. 2026 May 27;16(6):e73691. doi: 10.1002/ece3.73691 (PMC13239910; doi:10.1002/ece3.73691)

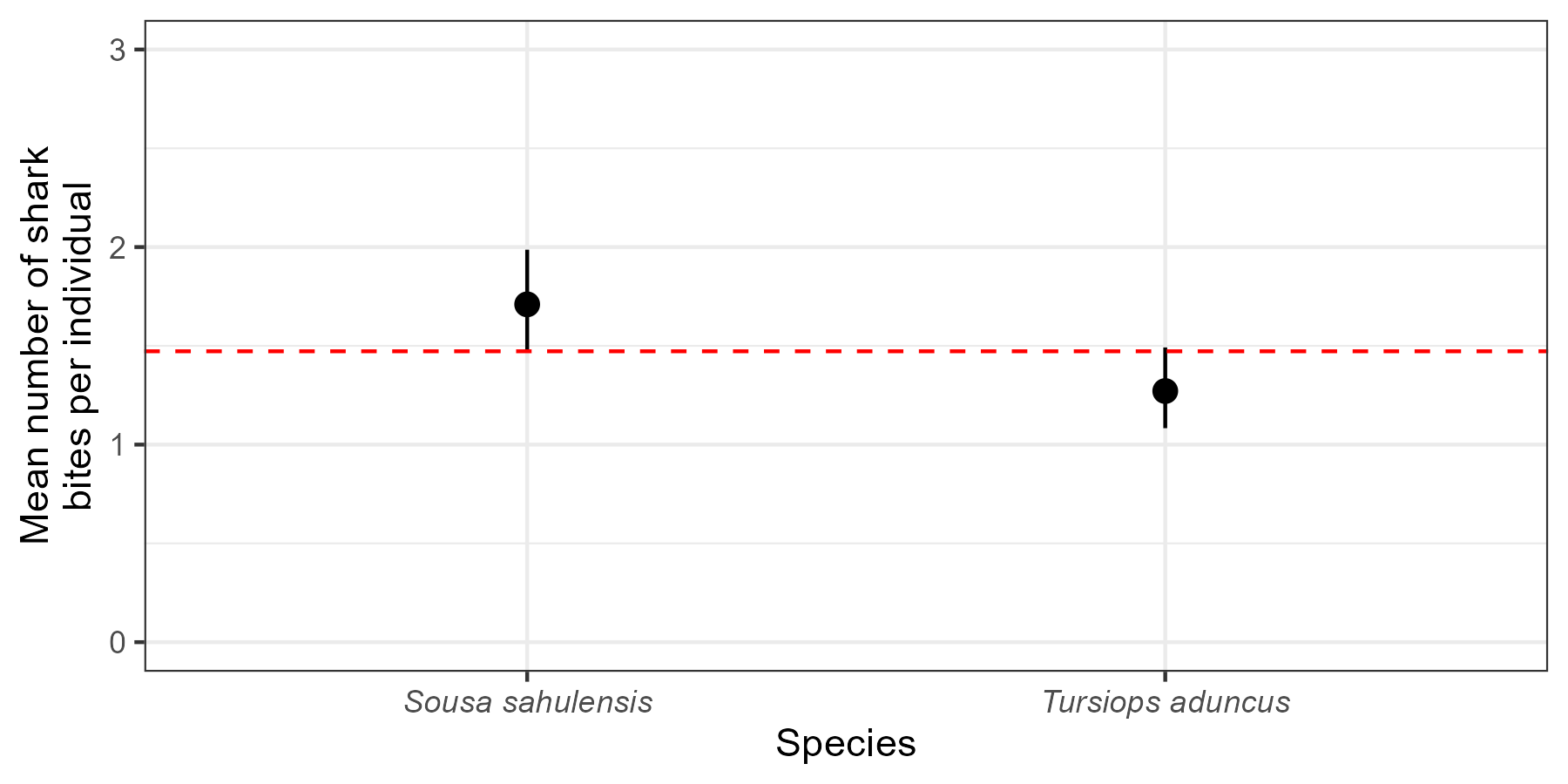

Supplement: Supplementary file 1 — Figure S1: Mean number of shark bites per individual observed on bitten Sousa sahulensis and Tursiops aduncus in southeast Queensland. Red dotted line denotes the mean number across all species, error bars are shown. [file ECE3-16-e73691-s002.tiff]
