# Supplementary material for: Investigating the Predation Risk of Coastal Dolphins via the Presence of Shark Bite Scars Across Southeast Queensland, Australia
Source: Ecol Evol. 2026 May 27;16(6):e73691. doi: 10.1002/ece3.73691 (PMC13239910; doi:10.1002/ece3.73691)
Supplement: Supplementary file 2 — Table S1: Model selection by AICc, ΔAICc (for models < 2), AICc weights and the log‐likelihood of the top models for shark bite presence and the number of bites for dolphin species in southeast Queensland, for only individuals that were photographed on both sides. Table S2: Poisson generalised linear model (GLM) results of the top‐ranked model (predictor: species) of the number of shark bites per individual on dolphins in southeast Queensland. The reference species is S. sahulensis . Significance denoted in bold. [file ECE3-16-e73691-s001.docx]

SUPPORTING INFORMATION

| Table S1: Model selection by AICc, ΔAICc (for models <2), AICc weights and the log-likelihood of the top models for shark bite presence and the number of bites for dolphin species in south-each Queensland, for only individuals that were photographed on both sides. | | | | | | |
| --- | --- | --- | --- | --- | --- | --- |
| Model | **Predictors** | **df** | **AICc** | **ΔAICc** | **ωAIC** | **log-lik** |
| Bite presence (Binomial GLM) | Sharkbite ~ Species + Age.Class + Habitat_type + Age.Class:Habitat_type | 5 | 638.9 | 0.00 | 0.18 | -321.12 |
|  | Sharkbite ~ Species + Age.Class + Habitat_type + Age.Class:Habitat_type + Species:Habitat_type | 6 | 640.7 | 1.79 | 0.07 | -314.28 |
|  | Sharkbite ~ Species + Age.Class + Habitat_type + AvgGroupSize + Age.Class:Habitat_type + Age.Class:Species | 6 | 640.8 | 1.86 | 0.07 | -314.31 |
|  | Sharkbite ~ Species + Age.Class + Habitat_type | 4 | 640.8 | 1.86 | 0.07 | -316.36 |
|  | Sharkbite ~ Species + Age.Class + Habitat_type + AvgDepth + Age.Class:Habitat_type | 6 | 640.8 | 1.91 | 0.07 | -314.34 |
|  | Sharkbite ~ Species + Age.Class + Habitat_type + AvgGroupSize + Age.Class:Habitat_type | 6 | 640.9 | 1.93 | 0.07 | -314.35 |
| Count of bites (Poisson GLM) | Number_of_bites ~ Species | 2 | 565.8 | 0.00 | 0.10 | -280.85 |
|  | Number_of_bites ~ Species + AvgGroupSize | 3 | 566.0 | 0.29 | 0.09 | -279.97 |
|  | Number_of_bites ~ Species + Age.Class | 3 | 566.2 | 0.47 | 0.08 | -280.06 |
|  | Number_of_bites ~ Species + AvgGroupSize + Age.Class | 4 | 566.7 | 0.96 | 0.06 | -279.27 |
|  | Number_of_bites ~ Species + AvgDepth | 3 | 567.7 | 1.92 | 0.04 | -280.79 |
|  | Number_of_bites ~ Species + Habitat_type | 3 | 567.7 | 1.93 | 0.04 | -280.79 |

| Table S2: Poisson generalised linear model (GLM) results of the top-ranked model (predictor: species) of the number of shark bites per individual on dolphins in south-east Queensland. The reference species is *S. sahulensis*. Significance denoted in bold*.* | | | | | |
| --- | --- | --- | --- | --- | --- |
| Model | **Predictor** | **Estimate (β)** | **SE** | ***z*-value** | ***p*-value** |
| Number of bites | Intercept | 0.54 | 0.08 | 7.02 | **<0.001** |
|  | Species (*T. aduncus*) | -0.30 | 0.11 | -2.65 | **<0.05** |


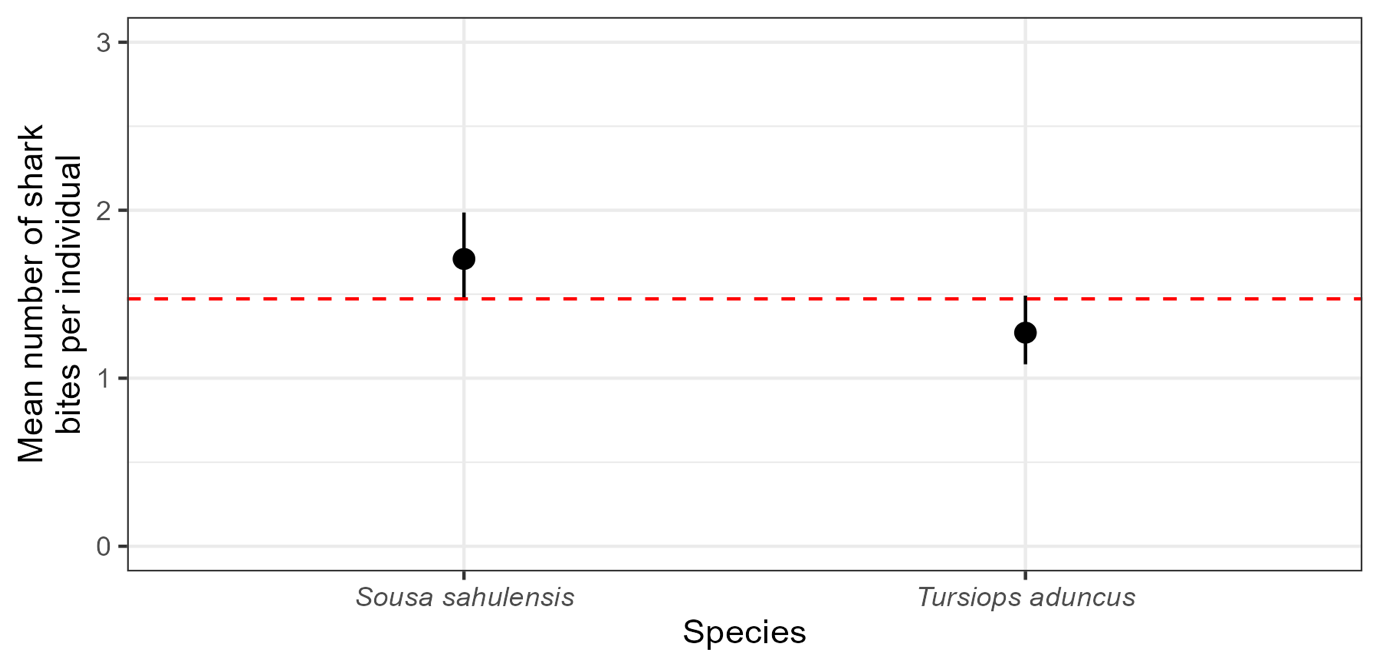


**FIGURE S1:** Mean number of shark bites per individual observed on bitten *Sousa sahulensis* and *Tursiops aduncus* in southeast Queensland. Red dotted line denotes the mean number across all species, error bars are shown.
